# Retrieval of functional TCRs from single antigen-specific T cells: Toward individualized TCR-engineered therapies

**DOI:** 10.1080/2162402X.2015.1005523

**Published:** 2015-03-19

**Authors:** Tana Omokoko, Petra Simon, Özlem Türeci, Ugur Sahin

**Affiliations:** 1 TRON – Translational Oncology at the University Medical Center of Johannes Gutenberg University; Mainz, Germany; 2 BioNTech Cell & Gene Therapies GmbH; Mainz, Germany; 3 Research Center for Immunotherapy (FZI) at the University Medical Hospital; Johannes Gutenberg-University; Mainz, Germany

**Keywords:** adoptive cell transfer, epitope, personalized immunotherapy, T cell engineering, T cell receptor

## Abstract

We have developed a highly versatile platform for the systematic retrieval of T-cell receptors (TCRs) from single-antigen-reactive T cells and for characterization of their function and specificity. This approach enables rapid extraction of multiple TCRs from repertoires in individuals and not only broadens the diversity of TCRs suitable for clinical use, but also sets the stage for actively personalized immunotherapeutic strategies.

## Introduction

The tailored reprogramming of immune cells from cancer patients to recognize and attack their tumors has potential as a disruptive medical innovation.^1^ Reports of impressive clinical responses in patients with advanced hematologic malignancies have offered a glimpse of the power of immune–receptor-engineered T cells redirected against cancer cells.^2^ Exploitation of the full potential of this technology, however, is hindered by the paucity of suitable immune receptors targeting antigens that are selectively expressed in tumor cells. The array of T-cell receptors (TCRs) currently available for safe clinical use is directed against a few well-known tumor antigens and restricted to human leukocyte antigen (HLA)–A2-presented epitopes.^3^

The pool of antigens potentially suitable as immunotherapeutic targets for TCR-engineered T cells has grown considerably. However, for the vast majority of these antigens, neither TCRs nor T-cell clones have been defined as yet.

As a consequence, there is an ample need for a technology enabling the systematic recovery of sets of functional TCRs against defined antigens from individual repertoires as well as swift definition of their specificity.

**Figure 1.**
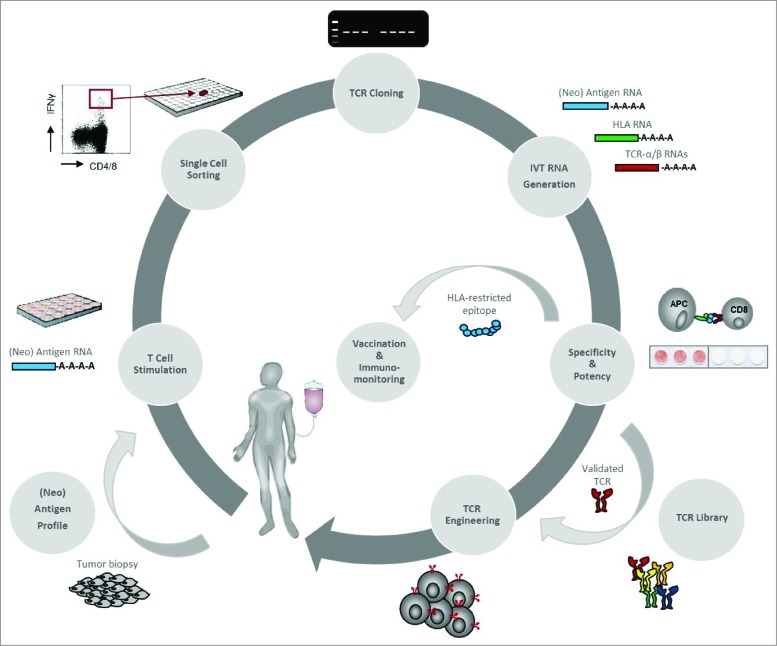
Discovery and validation of antigen-specific TCRs from single T cells and its use for personalized immunotherapy. For personalized TCR gene therapy, the target expression profile of the patient's tumor is analyzed with regard to shared or mutated tumor-specific antigen expression by RT-PCR and next-generation sequencing (NGS)-based methods, respectively. T cells of the patient are stimulated with autologous dendritic cells (DCs) transfected with (neo)antigen-encoding *in vitro*-transcribed RNA (IVT-RNA). Antigen-specific CD4^+^ or CD8^+^ T cells are isolated by flow cytometry based on induced upregulation of activation markers or cytokine secretion. Single cells are harvested in multi-well plates for RNA extraction. Full-length TCRα/β V(D)J regions are amplified and directly cloned into vectors containing TCRα/β constant region cassettes for subsequent *in vitro* transcription. To test TCR specificity and potency, T cells are co-transfected with IVT-RNA encoding the corresponding TCRα/β chains. As antigen-presenting cells, either autologous DCs or K562 cells expressing the respective antigen–HLA combination after IVT-RNA transfer are used. Validated TCRs are used for genetic engineering of the patient's autologous T cells and generation of a library providing TCR reagents for patients with appropriate HLA and tumor antigen expression.

Recently, we presented a platform capable of fulfilling this need (**Fig. 1**). The workflow is initiated by stimulation of the T cells of donors with autologous antigen-presenting cells (APCs) transfected with *in vitro*-transcribed synthetic RNA encoding a defined antigen of interest.^4^ Antigen-specific CD4^+^ and CD8^+^ T cells are identified by assessing activation markers and then isolated by cell sorting. The TCRα and -β chains are amplified from these single T lymphocytes by reverse transcription polymerase chain reaction (RT-PCR) using oligonucleotide primer sets designed to cover all TCR gene families. For functional characterization, T cells are transfected with synthetic RNA to express the identified TCRα/β pairs and are assessed by using target cells that are reconstituted for the expression of the respective antigen in conjunction with the donor's HLA alleles.

We established this platform using the cytomegalovirus (CMV) phosphoprotein pp65 as a model antigen. We obtained 13 different CMV–pp65-specific TCRs recognizing HLA class I and II restricted epitopes, both known and novel ones.

For further proof of concept, we resorted to NY-ESO-1, one among the immunologically best-characterized members of the cancer/germline antigen family. NY-ESO-1 is known to frequently elicit spontaneous CD4^+^ and CD8^+^ T-cell responses in patients with cancer, the specificities of which have been mapped over the last few years. Sixteen NY-ESO-1–specific TCRs were isolated from 3 seropositive patients with non–small cell lung cancer (NSCLC). These were directed against 11 different HLA class-I and -II restricted epitopes, again known and novel ones, clustering in known immunogenic regions of the NY-ESO-1 protein.

Finally, we applied this TCR identification approach to the transmembrane phosphatase with tensin homology (TPTE), which belongs to a large group of antigens with high cancer cell selectivity, for which spontaneous T-cell responses have not yet been reported in cancer patients. TPTE-specific T cells were isolated from 3 seropositive patients with NSCLC, and a total of 27 TPTE-specific TCRs were cloned. Thus, multiple HLA class-I and -II restricted epitopes distributed over the whole protein sequence were identified, none of which had been identified earlier.

In summary, we identified 398 TCRs representing 189 different clonotypes. Among these, 56 TCRs were shown to be antigen specific corresponding to approximately one third of the uniquely identified TCRs. Notably, the majority of specificities have not been described earlier, although 2 of the antigens have been extensively studied as T-cell antigens for decades now. Moreover, the majority of TCRs were shown to recognize HLA class-II restricted epitopes, whereas the currently available and clinically explored TCRs are all HLA class-I restricted epitopes. The CD4^+^ T cells are of particular interest, as coadministration of CD4^+^ T cells to TCR-engineered adoptively administered T cells has been shown to enhance tumor infiltration of CD8^+^ T cells and to prevent their exhaustion.

The single-cell TCR isolation technology is versatile and can be adapted to clone human TCRs from different sources such as *in vitro*-sensitized lymphocytes or *in vivo*-primed T cells obtained from cancer patients. T cells from peripheral blood or from infiltrates in tumor lesions may also be used as a TCR source.

This technology can be envisioned to foster 3 clinical treatment concepts:

1. It can be applied to identify single TCRs of interest against defined antigen–HLA combinations to be developed for stratified approaches.
2. As the discovery process is efficient and the output is high, the technology allows setting up a warehouse of TCRs against multiple antigens and various HLA restrictions to accommodate personalized approaches. The TCR matching each individual patient's tumor and HLA haplotype would be chosen “off the shelf.” As studies suggest a benefit of targeting 2 or more antigens and simultaneously addressing HLA class I and II epitopes to circumvent tumor escape and achieve optimal T-cell effector functions, combinations of TCRs could be customized from such TCR warehouses.
3. The rapidity of obtaining TCRs confers the power to enter the new dimension of actively individualized approaches.

Cloning and functional immunological characterization of TCRs from 1 individual is accomplished in less than 2 weeks, and the platform is operated by liquid handling stations and a barcode-assisted sample tracking and documentation system. TCRs of individual patients can be defined and manufactured on-demand for “just-in-time” customized, engineered T cells for adoptive transfer. This is particularly attractive in conjunction with cancer-specific mutations as immunotherapy targets. The first evidence of the spontaneous T-cell responses directed against mutated gene products in cancer patients was generated in the 1990s.^5^ Meanwhile, there is broad acceptance of the enormous potential of mutation-specific T cells to confer antitumor activity in cancer patients.^6-9^ Due to the uniqueness of the repertoire of mutations (“the mutanome”) in every patient's tumor,^1^ they can only be exploited for patient-tailored approaches. We have recently proposed a personalized immunotherapy tapping the spectrum of individual mutations with tailored vaccines.^10^ An analogous personalized immunotherapeutic approach could be pursued with TCR-engineered T cells. Autologous TCRs re-directing T cells against such neo-epitopes are expected to exhibit an ideal efficacy and safety profile. As they are selected *in vivo*, they should have a sufficient affinity conferring sensitive recognition of the respective mutation. Their mutation specificity excludes on-target toxicity. Moreover, the negative selection in the autologous host minimizes the risk of off-target toxicity.

In summary, the single-cell TCR isolation approach presented here has the capability to broaden the range of patients and malignancies eligible for TCR-engineered immunotherapies and opens up avenues for personalized TCR-engineered treatment concepts.

## References

[1] Kershaw MH, Westwood JA, Darcy PK. Gene-engineered T cells for cancer therapy. Nat Rev Cancer 2013; 13:525–41; PMID:; http://dx.doi.org/10.1038/nrc3565 23880905

[2] Porter DL, Levine BL, Kalos M, Bagg A, June CH. Chimeric antigen receptor-modified T cells in chronic lymphoid leukemia. N Engl J Med 2011; 365:725–33; PMID:; http://dx.doi.org/10.1056/NEJMoa1103849 21830940 PMC3387277

[3] Hinrichs CS, Restifo NP. Reassessing target antigens for adoptive T-cell therapy. Nat Biotechnol 2013; 31:999–1008; PMID:; http://dx.doi.org/10.1038/nbt.2725 24142051 PMC4280065

[4] Simon P, Omokoko TA, Breitkreuz A, Hebich L, Kreiter S, Attig S, Konur A, Britten CM, Paret C, Dhaene K, et al. Functional TCR retrieval from single antigen-specific human T cells reveals multiple novel epitopes. Cancer Immunol Res 2014; 2:1230–44; PMID:; http://dx.doi.org/10.1158/2326-6066.CIR-14-0108 25245536

[5] Wolfel T, Hauer M, Schneider J, Serrano M, Wölfel C, Klehmann-Hieb E, De Plaen E, Hankeln T, Meyer zum Büschenfelde KH, Beach D. A p16INK4a-insensitive CDK4 mutant targeted by cytolytic T lymphocytes in a human melanoma. Science 1995; 269:1281–4; PMID:; http://dx.doi.org/10.1126/science.7652577 7652577

[6] Gubin MM, Zhang X, Schuster H, Caron E, Ward JP, Noguchi T, Ivanova Y, Hundal J, Arthur CD, Krebber WJ, et al. Checkpoint blockade cancer immunotherapy targets tumour-specific mutant antigens. Nature 2014; 515:577–81; PMID:; http://dx.doi.org/10.1038/nature13988 25428507 PMC4279952

[7] Snyder A, Makarov V, Merghoub T, Yuan J, Zaretsky JM, Desrichard A, Walsh LA, Postow MA, Wong P, Ho TS, et al. Genetic basis for clinical response to CTLA-4 blockade in melanoma. N Engl J Med 2014; 371:2189–99; PMID:; http://dx.doi.org/10.1056/NEJMoa1406498 25409260 PMC4315319

[8] Tran E, Turcotte S, Gros A, Robbins PF, Lu YC, Dudley ME, Wunderlich JR, Somerville RP, Hogan K, Hinrichs CS, et al. Cancer immunotherapy based on mutation-specific CD4+ T cells in a patient with epithelial cancer. Science 2014; 344:641–5; PMID:; http://dx.doi.org/10.1126/science.1251102 24812403 PMC6686185

[9] Matsushita H, Vesely MD, Koboldt DC, Rickert CG, Uppaluri R, Magrini VJ, Arthur CD, White JM, Chen YS, Shea LK, et al. Cancer exome analysis reveals a T-cell-dependent mechanism of cancer immunoediting. Nature 2012; 482:400–4; PMID:; http://dx.doi.org/10.1038/nature10755 22318521 PMC3874809

[10] Castle JC, Kreiter S, Diekmann J, Löwer M, van de Roemer N, de Graaf J, Selmi A, Diken M, Boegel S, Paret C, et al. Exploiting the mutanome for tumor vaccination. Cancer Res 2012; 72:1081–91; PMID:; http://dx.doi.org/10.1158/0008-5472.CAN-11-3722 22237626

